# Hair regrowth in a patient with central centrifugal cicatricial alopecia after a 2-month trial of baricitinib

**DOI:** 10.1016/j.jdcr.2023.07.016

**Published:** 2023-07-28

**Authors:** Kaelynn Workman, Chesahna Kindred

**Affiliations:** a Case Western Reserve University School of Medicine, Cleveland, Ohio; b Kindred Hair & Skin Center, Columbia, Maryland

**Keywords:** alopecia, baricitinib, CCCA, central centrifugal cicatricial alopecia, hair loss, JAK-inhibitors, scarring alopecia

## Introduction

Central centrifugal cicatricial alopecia (CCCA) is a primary lymphocytic cicatricial alopecia that is common in African-American women.^1^ It typically presents as a singular alopecic patch in the crown of the head that slowly expands and progresses centrifugally, with surrounding broken hairs.^1^ Historically, CCCA is thought to be caused by genetic susceptibility triggered by certain hair styling methods like thermal and chemical hair straightening along with high traction hairstyles.^1^ Recent genetic studies suggest a variant of the gene *PADI3*, which encodes a protein that posttranslationally modifies other proteins involved in hair shaft formation, may be implicated in the cause of this disease.^2^ Due to the pathogenesis of this disease being much less understood than other forms of alopecia, treatment options remain limited. Topical metformin has been reported to show significant success due to its antifibrotic properties, as fibrosis is strongly implicated in the pathogenesis of this disease.^3^^,^^4^ While fibrosis is strongly implicated in the pathogenesis of CCCA, much of the treatment surrounding CCCA is based on reducing the inflammatory response.^1^ Janus Kinase (JAK) inhibitors are effective pharmaceuticals for controlling overactive immune and inflammatory pathways through inhibition of the JAK/signal transducer and activator of transcription (STAT) pathway.^5^ Here, we report a case of CCCA that showed significant hair regrowth after a two-month-long trial of baricitinib.

## Case report

**Fig 1:**
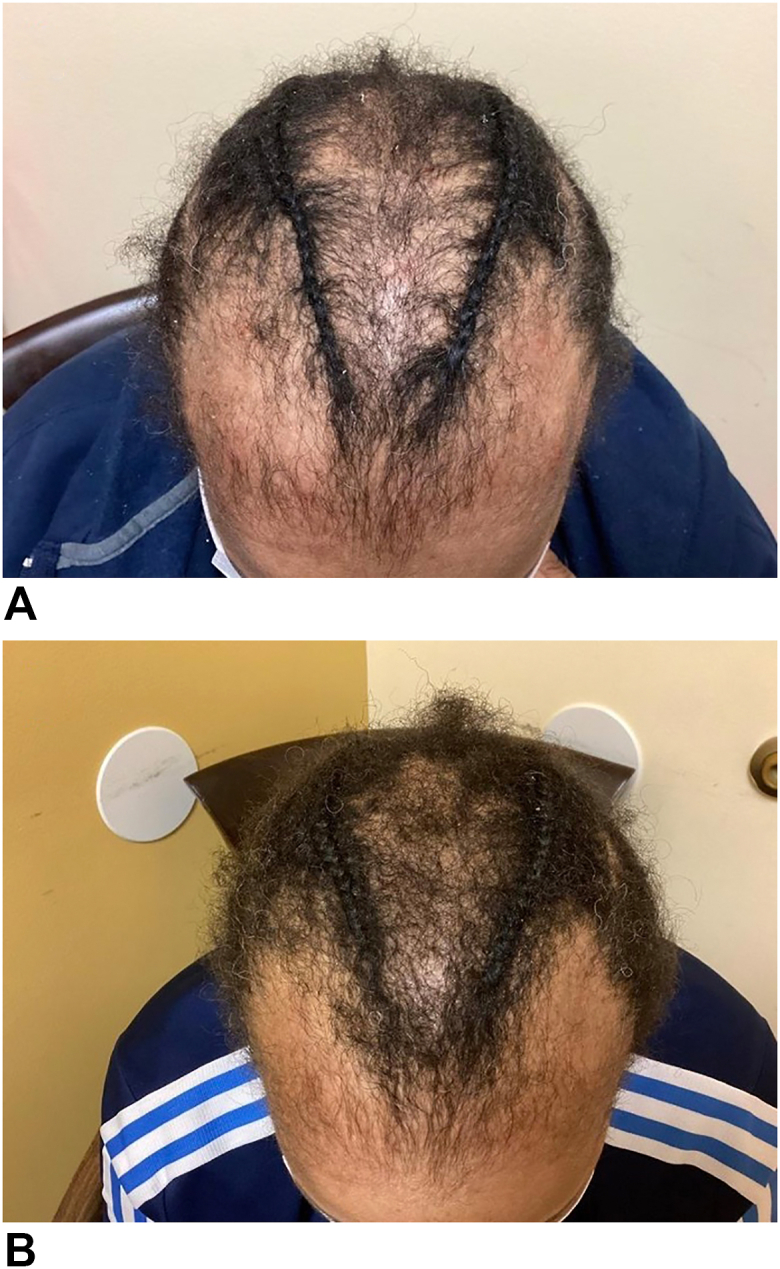
Hair regrowth in Central Centrifugal Cicatricial Alopecia in a patient on a JAK-inhibitor. **A,** CCCA 1-month poststart of baricitinib. **B,** CCCA 2-month poststart of baricitinib. This patient was started on a 2-month long trial of baricitinib for treatment of her CCCA without reporting of any major negative side effects caused by baricitinib. Significant hair growth can be seen along the frontal and vertex scalp between the 2 braids and also along the parietal scalp adjacent to the braids. Further, both braids appear to be thicker.

A 42-year-old African-American woman presented to an outpatient dermatology practice with a 5-year history of alopecia accompanied by burning sensation and itching of the scalp. Two biopsies were performed and revealed end-stage cicatricial alopecia and early changes of androgenetic alopecia (AGA) involving the frontal scalp and CCCA involving the medial parietal scalp. Histopathological findings of the frontal scalp showed marked reduction in the number of hairs with true follicular scaring, hair miniaturization, and mild chronic perifollicualr inflammation. In the parietal scalp biopsy, there was a marked reduction in the number of hairs, destruction of sebaceous glands, no evidence of miniaturization, premature desquamation of the inner root sheath, and mild-to-moderate chronic perifollicular inflammation. For the past two and a half years, she was managed at the same practice. Her treatment consisted of 40 mg of oral doxycycline daily, 2.5 mg of oral minoxidil daily, 0.1% topical solution of halcinonide twice daily as needed for symptoms of inflammation, and monthly 5 mg/mL of triamcinolone intralesional triamcinolone injections. The only significant past medical history for the patient was asthma. After several months, the CCCA became stable. However, the disease repeatedly worsened when the intralesional cortisone injections were extended beyond once a month. Higher doses of doxycycline caused intolerable gastrointestinal symptoms. Two years after the initial presentation, the symptoms of inflammation returned despite treatment adherence. Off-label use of baricitinib 4 mg was initiated and the patient was continued on monthly intralesional triamcinolone injections. Both 0.1% topical halcinonide and 40 mg of oral doxycycline were discontinued. Laboratory examinations taken before the start of baricitinib were within normal limits. After 1 month, the symptoms resolved. After 2 months, regrowth in the medial parietal scalp with biopsy-proven CCCA was noted. No significant regrowth was noted along the bifrontal scalp with biopsy-proven AGA. Pictures were taken at month 1 (Fig 1, *A*) and month two (Fig 1, *B*) poststarting baricitinib for documentation.

## Discussion

CCCA is a scarring alopecia that is not well researched and is very difficult to treat, often leaving the patient with very few options for treatment. Despite this, multiple anti-inflammatory pharmaceuticals and immuno-modulating therapies have been tried in the treatment of this disease with varied results.^1^ Further, studies investigating the role of STAT3 in lymphocytes have shown that STAT3 is activated in the perifollicular lymphocytes in patients with CCCA.^6^ Activation of STAT3 is significant for increasing Th17 cells, which are responsible for secreting pro-inflammatory cytokines that could play a role in the development in this disease. JAK-inhibitors, a class of small molecule enzyme inhibitors, inhibit the JAK-STAT pathway by binding to the ATP binding pocket on the JAK.^5^ This prevents ATP from binding and thus prevents the JAK from carrying out its function of phosphorylation. While no JAK-inhibitor has complete specificity for a particular JAK, every JAK-inhibitor has greater specificity for certain JAKs than others. In this case specifically, baricitinib is more selective for JAK 1 and 2.^5^ As of recent, JAK-inhibitors have been trialed and studied in the treatment of alopecia areata, a nonscarring alopecia caused by the attack of the hair follicle by the immune system.^7^ Due to the possible implication of STAT involvement in the pathogenesis of CCCA, this patient was trialed on baricitinib with significantly positive results. While the exact role JAK-inhibitors play in the pathogenesis of CCCA is still unclear, the positive results seen in this case offer potential insights into treatment options for this disease. While this report increases potential insights into treatment options available for CCCA patients, it also increases insights into the pathogenesis of CCCA and the role the JAK-STAT pathway may play in this form of alopecia. Further research is needed to elucidate the role of JAKs in CCCA.

## Conflicts of interest

CK: Lilly: advisory board and speaker. UCB: advisory board and speaker. Aerolase: board member and speaker. Nutrafol: speaker. Novartis: advisory board. Sun Pharmaceuticals: advisory board and speaker. AAD Web content reviewer. Journal of the National Medical Association: editor. Cutis: journal editor. Janssen: steering committee, SOC Advisory Board. Abbvie-consultant. Pfizer- speaker. Regeneron - advisory board and speaker.
